# Cardiometabolic Comorbidities in COPD: Focus on Diabetes, GLP-1 Receptor Agonists, SGLT-2 Inhibitors and Antidiabetic Drugs

**DOI:** 10.3390/jcm15052082

**Published:** 2026-03-09

**Authors:** Maria Kallieri, Georgios Hillas, Stelios Loukides, Konstantinos Kostikas, Athena Gogali

**Affiliations:** 1 2nd Respiratory Medicine Department, “Attikon” University Hospital, National and Kapodistrian University of Athens, 12462 Athens, Greece; mkallieri@yahoo.gr (M.K.); loukstel@med.uoa.gr (S.L.); 2 Respiratory Medicine Department, University of Ioannina, 45110 Ioannina, Greece; ktkostikas@gmail.com (K.K.);

**Keywords:** COPD, type 2 diabetes, antidiabetic drugs, GLP-1RAs, SGLT-2 inhibitors

## Abstract

**Background/Objectives:** The coexistence of chronic obstructive pulmonary disease (COPD) and type 2 diabetes mellitus (T2D) poses significant clinical challenges due to overlapping mechanisms of systemic inflammation, oxidative stress, hypoxia, and metabolic dysregulation. Patients with both conditions face higher risks of exacerbations, prolonged hospitalizations, cardiovascular events, and reduced quality of life. This review aims to summarize current evidence on the pathophysiological interplay between COPD and T2D and to evaluate the impact of lifestyle and pharmacologic interventions. **Methods:** A narrative review of the literature was conducted to evaluate the pathophysiological links between COPD and T2D, assess the effects of pharmacologic and lifestyle interventions, and highlight key gaps and priorities for future research, with an emphasis on integrated, evidence-based management for this high-risk population. **Results:** Lifestyle interventions, including smoking cessation and structured physical activity, remain foundational to management. Emerging evidence indicates that antidiabetic therapies, such as glucagon-like peptide-1 receptor agonists (GLP-1RAs) and sodium–glucose cotransporter-2 inhibitors (SGLT-2is), may confer additional pulmonary, metabolic, and cardiovascular benefits. These agents modulate systemic inflammation, oxidative stress, endothelial function, and insulin sensitivity, potentially reducing COPD exacerbations, improving lung function, and enhancing survival. Safety concerns, including glucocorticoid-induced hyperglycaemia and hypoxia-related metabolic complications, underscore the need for careful monitoring and individualized therapy COPD patients. **Conclusions:** Optimal care requires a multidisciplinary, patient-centred approach integrating pulmonology, endocrinology, primary care, nutrition, and rehabilitation, alongside shared decision-making and patient education. Despite promising findings, critical knowledge gaps remain. Large, well-designed randomized controlled trials and standardized definitions are needed to guide personalized therapeutic strategies.

## 1. Introduction

Chronic obstructive pulmonary disease (COPD), a heterogeneous lung disease characterized by persistent respiratory symptoms, such as dyspnoea, cough and sputum production, is one of the most common chronic respiratory conditions in adults and it is frequently associated with a variety of comorbid diseases [1]. Among these, metabolic syndrome (MetS) has attracted increasing attention due to its clinical and prognostic implications. MetS is a cluster of conditions that includes central obesity, insulin resistance, hypertension, dyslipidaemia and impaired glucose tolerance, which increases the risk of developing cardiovascular disease (CVD) and type 2 diabetes (T2D). T2D, itself, is among the most prevalent comorbidities of COPD, affecting approximately 30–40% of COPD patients [2]. Patients with both T2D and COPD have an increased risk of COPD exacerbations, increased healthcare utilization and hospital admissions, and are more likely to experience non-respiratory complications [3]. Recent studies have shown that patients with both conditions have a disproportionately higher risk of cardiovascular disease and mortality than those with either disease alone, while observational studies have associated hyperglycaemia with accelerated lung function decline and adverse respiratory outcomes [4,5].

The close relationship between COPD and T2D may be explained by their shared risk factors and overlapping pathophysiological mechanisms, including chronic systemic inflammation, oxidative stress, physical inactivity, and tissue hypoxia [6]. Additionally, certain pharmacological treatments for COPD, particularly systemic corticosteroids, are known to exacerbate hyperglycaemia and increase the risk of developing T2D, further complicating disease management, while antidiabetic drugs, such as glucagon-like peptide-1 receptor agonists (GLP-1RAs) and sodium–glucose cotransporter-2 inhibitors (SGLT-2is), may exert beneficial effects beyond glycaemic control, potentially improving pulmonary outcomes and systemic inflammation in patients with COPD [7,8,9,10,11,12]. These findings have caused growing interest in exploring the relationship between COPD and T2D and the potential therapeutic implications of antidiabetic agents in patients with chronic respiratory disease.

The aim of the current review is to explore the link between COPD and T2D, and to evaluate the emerging evidence on the potential therapeutic role of antidiabetic agents in improving clinical outcomes and metabolic control among patients with COPD.

## 2. Methodology

A structured literature search was performed using the Pubmed database https://pubmed.ncbi.nlm.nih.gov/ up to 23 February 2026, utilizing Medical Subject Heading (MeSH) and free-text terms such as “Chronic obstructive pulmonary disease”, “COPD”, “type 2 diabetes”, “T2D”, “T2DM”, “SGLT-2 inhibitors”, “GLP-1RAs”, “metformin”, “inflammation”, “hypoxia”, and “exacerbations”, as well as related keywords and their combinations. Reference lists of relevant articles and reviews were also manually screened to identify additional eligible studies. Eligible articles included preclinical experimental studies, observational clinical studies, randomized or non-randomized interventional clinical trials, published in peer-reviewed journals that examined the association or shared pathophysiological mechanisms between the two conditions, such as inflammation, oxidative stress, or metabolic dysfunction, or assessed the effects of antidiabetic drugs on pulmonary outcomes. In addition, systematic reviews and meta-analyses providing mechanistic or therapeutic insights into the comorbidity of COPD and T2D were also included to ensure a comprehensive understanding of the topic. Non-English studies, case reports, editorials and studies lacking sufficient data were not included. Given the narrative nature of the review, formal risk-of-bias assessment was not performed; however, study design and methodological quality were considered when interpreting the strength of evidence.

A total of 127 articles were included in the final narrative synthesis. These comprised preclinical/experimental studies, observational clinical studies, randomized or interventional clinical trials, and systematic reviews or meta-analyses, reflecting the heterogeneous nature of the available evidence on COPD and T2D and their shared therapeutic targets.

Included studies were stratified according to study design into four predefined categories: preclinical/experimental studies (animal or cellular mechanistic investigations, *n* = 33), observational clinical studies (cohort, case–control, registry, Mendelian randomization, and pharmaco-epidemiological studies, *n* = 32), randomized or interventional clinical trials (*n* = 2), and systematic reviews or meta-analyses (*n* = 60). This stratification was used to contextualize the strength and limitations of the available evidence discussed throughout the review.

## 3. Epidemiology

A systematic review of 4208 patients with COPD showed that more than one third of the patients had MetS (~34%) [13]. Moreover, compared to controls, the proportion of COPD patients with MetS was significantly higher (32% vs. 30%, *p* = 0.001), with arterial hypertension (56% vs. 51%), abdominal obesity (39% vs. 38%) and hyperglycaemia (44% vs. 47%) being the most common in both groups [1]. In a study involving 76 participants, 42% were found to have MetS, and these individuals experienced exacerbations more frequently than those without the condition [14].

In a meta-analysis of four cohort and three case–control studies, with 1,369,560 participants, the risk of developing T2D was higher among patients that belonged to the COPD group [Odds Ratio (OR) = 1.17, 95% Confidence Interval (CI) 1.01–1.35, *p* = 0.03; I^2^ = 0%] [15]. However, when limited to studies using guideline-based diagnoses for both COPD and T2D, the association was not statistically significant (OR = 1.17, 95% CI 0.96–1.42, *p* = 0.12) [15]. What is more, there are data that suggest that impaired lung function correlates with increased risk of T2D [15]. Specifically, in a meta-analysis of thirteen cohort studies that included 7,683,784 participants, patients with COPD had a higher risk of T2D Relative Risk (RR) = 1.25, 95% CI 1.16–1.34), and people in the lowest category of forced vital capacity as % predicted (FVC%) had a 43% higher risk of developing T2D compared with those in the highest category (RR = 1.43, 95% CI 1.33–1.53). Likewise, those in the lowest category of forced expiratory volume in 1 s as % predicted (FEV_1_%) had a 49% higher risk of T2D compared with those in the highest category (RR = 1.49, 95% CI 1.39–1.60) [16].

Several studies have evaluated the incidence and prognosis of T2D among patients hospitalized for acute exacerbations of COPD (AECOPD). The prevalence of T2D in this population is substantial, with reports indicating that approximately one-third of AECOPD patients may have diabetes or prediabetes. Comorbid T2D has been consistently associated with worse clinical outcomes, including longer hospital stays, higher healthcare costs, increased 30-day readmission rates, and elevated mortality rates [17,18,19].

## 4. Pathophysiological Interplay

### 4.1. Systemic Inflammation and Oxidative Stress

Low-grade inflammation is a key characteristic of both COPD and T2D. Both conditions are characterized by elevated circulating levels of pro-inflammatory cytokines, such as tumour necrosis factor-alpha (TNF-α), interleukin-6 (IL-6), and C-reactive protein (CRP), which play critical roles in promoting insulin resistance and deteriorating pulmonary function [20]. Chronic oxidative stress further amplifies tissue injury, perpetuating a cycle of metabolic and respiratory dysfunction that destabilizes homeostasis in both organ systems [20].

TNF-α, a cytokine known to be elevated in both conditions, disrupts insulin receptor signalling by promoting serine phosphorylation of insulin receptor substrate-1 (IRS-1), which reduces glucose uptake and systemic insulin resistance [21]. Furthermore, TNF-α enhances lipolysis, leading to increased circulating free fatty acids (FFAs) that exacerbate insulin resistance and stimulate hepatic gluconeogenesis, ultimately worsening glycaemic control in individuals with coexisting COPD and T2D [22,23].

IL-6, another key cytokine, augments hepatic glucose output and contributes to dyslipidaemia. IL-6 causes increased production of glucose in the liver and contributes to dyslipidaemia. Sustained IL-6 signalling has been associated with pancreatic β-cell dysfunction and apoptosis, leading to impaired insulin secretion and accelerating diabetes progression [23]. Moreover, IL-6 interferes with mitochondrial oxidative phosphorylation, amplifying oxidative stress-mediated injury in pulmonary and pancreatic tissues [24].

CRP, an acute phase protein that is elevated during inflammation, contributes to endothelial dysfunction, vascular stiffness and arterial inflammation by reducing nitric oxide (NO) bioavailability and accelerating atherosclerosis, thereby increasing cardiovascular risk in patients with COPD and T2D [25,26].

The chronic inflammation that characterizes patients with COPD causes a pro-inflammatory and anti-inflammatory mediators’ imbalance. Persistent activation of pathways such as nuclear factor-kappa B (*NF-κB*) and the NLR family pyrin domain containing 3 (*NLRP3*) inflammasome further contributes to systemic metabolic dysfunction, while the inhalation of cigarette smoke results in an increased production of reactive oxygen species (ROS), which causes oxidative stress and damages pancreatic β-cells, reducing insulin release and worsening hyperglycaemia [27,28]. COPD is also associated with increased levels of advanced glycation end-products (AGEs) that amplify oxidative stress and with their connection to the receptor for advanced glycation end-products (RAGE), activate pro-inflammatory cascades that injure both pulmonary tissue and pancreatic islets. Collectively, these interconnected mechanisms form a shared pathophysiological network linking COPD and T2D, which highlights the importance of targeting systemic inflammation and oxidative pathways to mitigate metabolic and respiratory decline in patients with dual disease burden [29].

It should be noted that most of the mechanistic evidence linking pathways such as *NLRP3* inflammasome activation, RAGE signalling, and *SIRT1* modulation to metabolic–pulmonary interactions comes from experimental and preclinical studies, and although these pathways provide important pathophysiological insights, their direct impact on clinically meaningful COPD outcomes in humans remains insufficiently established.

### 4.2. Hypoxia

Hypoxia, a condition frequently observed in patients with advanced COPD and chronic respiratory failure, plays a crucial role in impairing glucose metabolism and promoting insulin resistance through complex, interrelated mechanisms involving inflammation, mitochondrial dysfunction, and altered energy regulation.

Prolonged hypoxia leads to pancreatic β-cell loss, most commonly due to necrosis, but also through the activation of apoptotic pathways [30]. Evidence of caspase-3 colocalizing with hypoxia-inducible factor-1α (*HIF-1α*), a key protein involved in cellular regulation during hypoxia, within pancreatic islets, indicates that apoptosis may be concentrated in regions experiencing the greatest oxygen deprivation [31,32]. Sustained activation of *HIF-1α* has also been linked to increased hepatic gluconeogenesis and hyperglycaemia [33].

Chronic hypoxia also causes mitochondrial dysfunction and a reduction in ATP production, which reduces insulin sensitivity of skeletal muscle, while it contributes to cardiometabolic burden by promoting pulmonary hypertension, impairing vascular endothelial function, and disrupting erythropoiesis (28). These alterations amplify the likelihood of cardiovascular complications, which are already disproportionately common among individuals with coexisting COPD and T2D [34]. Furthermore, under hypoxic conditions, lipid metabolism becomes dysregulated, leading to enhanced lipolysis and accumulation of circulating free fatty acids. These metabolic disturbances aggravate insulin resistance, further linking hypoxia to impaired glucose homeostasis [35,36].

On the other hand, studies have explored the impact of oxygen supplementation on glucose metabolism in hypoxic COPD patients. A study found that providing supplemental oxygen to COPD patients with chronic hypoxaemia led to an immediate improvement in glucose tolerance [37]. Specifically, net peripheral glucose uptake increased by 29%, and tissue sensitivity to insulin improved by 32% (*p* = 0.03), highlighting the reversible nature of hypoxia-induced insulin resistance [37].

Furthermore, chronic intermittent hypoxia, as observed during obstructive sleep apnoea, a common comorbidity of COPD patients, contributes to MetS [38,39]. Sympathetic overactivation elevates catecholamine levels, driving hyperglycaemia, hyperinsulinemia, and impaired glucose uptake [38]. Additionally, intermittent hypoxia stimulates adipose-derived cytokines such as IL-6, TNF-α, and leptin, promoting lipolysis and free fatty acid release, which further exacerbates insulin resistance [40,41]. Animal models confirm that prolonged intermittent hypoxia leads to progressive hyperinsulinemia and glucose intolerance, highlighting its role in metabolic dysfunction [38,41].

### 4.3. Sedentary Lifestyle, Physical Inactivity and Sarcopenic Obesity

Sedentary behaviour (SB) is one of the most common modifiable risk factors for the development of T2D. It lacks a universally accepted definition but is commonly described as “any waking behaviour characterized by an energy expenditure ≤1.5 metabolic equivalents (METs), typically while sitting or reclining” [42]. Importantly, SB does not necessarily indicate an absence of physical activity (PA), rather, it reflects a predominance of low-energy activities throughout the day, such as working at a desk, driving, or watching television [43]. Large prospective studies, with more than a million participants, have shown that following a sedentary lifestyle is associated with increased risk of developing T2D [44]. In particular, a meta-analysis of 18 studies (16 prospective and 2 cross-sectional) including 794,577 participants showed that, compared with the lowest levels of sedentary time, the highest levels were associated with a more than two-fold increased risk of diabetes (RR 2.12; 95% CI 1.61–2.78), which was statistically significant [45]. Another meta-analysis of thirty-four studies (1,331,468 participants) found a linear association [RR 1.01 (1.00, 1.01)] independent of PA [44].

The importance of physical inactivity and sedentary behaviour is particularly pronounced in patients with COPD. Compared to age-matched healthy controls, COPD patients have reduced levels of physical activity, including shorter duration, lower intensity, and fewer daily activity counts [46]. This inactivity stems from disease-specific and behavioural barriers, with fear of breathlessness, advanced age, and diminished motivation being the most significant factors limiting engagement in regular physical activity [46].

SB and PA contribute to insulin resistance and the development of T2D through multiple interrelated mechanisms. Mitochondrial dysfunction represents a critical mechanistic link [47]. Mitochondria, as central regulators of cellular energy metabolism, are essential for effective insulin signalling and glucose homeostasis [47,48]. Physical inactivity and prolonged sedentary periods reduce mitochondrial content and impair the expression of key metabolic genes, including *PPARGC1A*, *CPT1B* (carnitine palmitoyltransferase 1B), and hexokinase II, leading to diminished oxidative capacity and increased oxidative stress in skeletal muscle [49,50,51].

Apart from mitochondrial dysfunction, studies have shown that physical inactivity upregulates subunits of NADPH oxidase (nicotinamide adenine dinucleotide phosphate oxidase) and elevates ROS, causing oxidative damage in vascular and muscle tissues in mice, which may lead to insulin resistance [52]. In addition, patients with reduced PA have higher markers of oxidative damage, such as malondialdehyde, and lower antioxidant defences, including superoxide dismutase, catalase, and glutathione peroxidase compared to active controls [53]. Together, these findings suggest that sedentary behaviour promotes oxidative stress, contributing to impaired peripheral insulin sensitivity, though further research is needed to fully elucidate these mechanisms.

Inactivity may also alter circulating sex hormones, including testosterone, oestradiol, and sex hormone-binding globulin (SHBG), reducing glucose transporter type 4 (Glut-4) translocation and downstream PI3K/Akt signalling, further compromising insulin sensitivity [6,54,55,56]. Additionally, reduced skeletal muscle capillarization limits nutrient and insulin delivery, while inactivity-induced accumulation of ceramides in muscle and adipose tissue exacerbates mitochondrial and endoplasmic reticulum stress, beta-cell dysfunction, and IRS-1 impairment [6]. Together, these factors demonstrate that a sedentary lifestyle disrupts metabolic homeostasis on multiple levels, highlighting the importance of physical activity in maintaining insulin sensitivity and preventing T2D.

**Figure 1:**
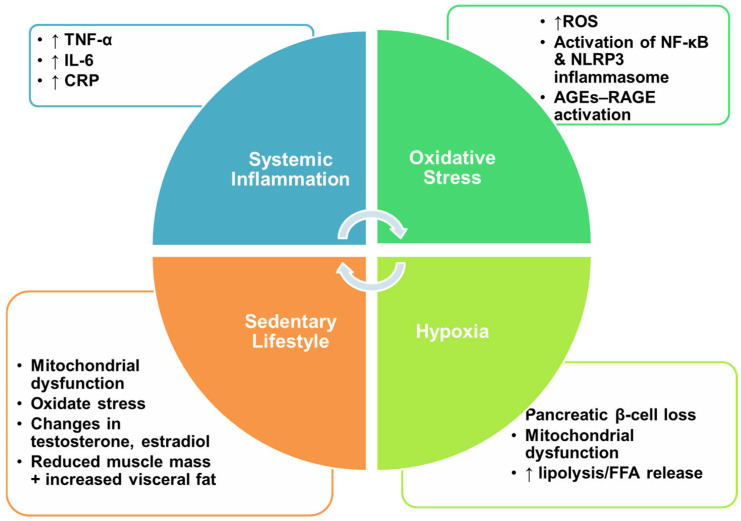
COPD-T2D pathophysiological interplay: Systemic inflammation, oxidative stress, sedentary lifestyle and hypoxia resulting in insulin resistance and deteriorating lung function. Abbreviations: TNF-α: Tumor necrosis factor-a, IL-6: Interleukine-6, CRP: C-reactive protein, ROS: Reactive oxygen species, *NF-κB*: Nuclear Factor kappa-light-chain-enhancer of activated B cells, *NLRP3*: NLR family pyrin domain containing 3, AGEs: Advanced glycation end-products, RAGE: Receptor of AGE, FFA: Free fatty acid, ↑: indicates increased levels.

Reduced physical activity in patients with COPD also accelerates muscle atrophy and loss of lean mass, and enhances the accumulation of visceral fat, leading to sarcopenic obesity. Sarcopenic obesity (SO), a clinical entity that combines sarcopenia and obesity, is a common comorbidity of COPD [57]. Patients with COPD in combination with SO have a more complex inflammatory profile, characterized by interactions between systemic inflammation and adipose tissue dysfunction that contribute to the development and progression of SO. Dysregulated adipocytokines, such as resistin, play key roles in lipid metabolism and exert pro-inflammatory effects. Specifically, resistin is secreted by human macrophages and causes insulin resistance by impairing the insulin PI3K-mTOR signalling pathway, which in turn reduces protein synthesis in skeletal muscle [58,59,60]. It may also inhibit myogenic differentiation of myoblasts, further compromising muscle function [60]. On the other hand, elevated inflammatory mediators, particularly TNF-α, further reflect systemic inflammation and are implicated as risk factors for skeletal muscle dysfunction [61]. Specifically, TNF-α activates TNF receptor 1, which suppresses AMP-activated protein kinase, causing accumulation of lipids in the muscle tissue, which ultimately leads to lipotoxicity-mediated insulin resistance [62,63]. Figure 1 summarizes COPD-T2D pathophysiological interplay that results in insulin resistance and deteriorating lung function.

## 5. Genetic Links

Emerging genetic evidence suggests a potential causal link between COPD and T2D. A recent two-sample Mendelian randomization (MR) analysis conducted in a European population revealed a modest but statistically significant causal effect of T2D on increased COPD risk (IVW OR: 1.002, 95% CI: 1.001–1.003, *p* = 0.001), a result further supported by MR-Egger sensitivity analysis (MR-Egger OR: 1.108, 95% CI: 1.016–1.208, *p* = 0.021) [64]. Interestingly, this relationship was not consistently replicated in Asian populations, suggesting that ethnic or genetic variations may influence disease susceptibility [64].

Conversely, a separate MR analysis using 26 independent single nucleotide polymorphisms (SNPs) strongly associated with COPD as instrumental variables, demonstrated that COPD may act as a risk factor for T2D, with an odds ratio of 1.06 (95% CI: 1.01–1.11, *p* = 0.006). Importantly, the results showed no indication of heterogeneity or horizontal pleiotropy, reinforcing the robustness of the findings [65]. Supporting this, a study from the Danish Twin Registry that included 13,649 twins indicated that shared genetic factors play a significant role in the comorbidity between COPD and T2DM, with a genetic correlation of 43% between the two conditions [66].

In addition to the existing MR evidence, further two-sample MR analyses have evaluated the genetic links between diabetes and COPD-related traits. One MR study found a marginal causal association between genetically predicted type 1 diabetes and increased COPD risk but did not identify a significant causal relationship for T2D in either direction, highlighting heterogeneity across diabetes subtypes and ancestries [67]. Another MR investigation examining diabetes and glycemic traits demonstrated significant associations between glycemic measures and pulmonary function (such as FEV_1_ and the FEV_1_/FVC ratio), suggesting complex genetic influences of metabolic traits on airflow limitation and respiratory health [68].

Collectively, these findings highlight a complex genetic interrelationship between T2D and COPD, supporting the concept of shared metabolic and inflammatory genetic pathways contributing to their coexistence.

## 6. Medication Effects

Systemic corticosteroids, administered orally or intravenously, remain a cornerstone in the short-term management of COPD exacerbations, with current guidelines recommending a dose equivalent to 40 mg of prednisolone daily for five days [1]. However, growing evidence highlights their significant metabolic adverse effects, particularly the risk of glucocorticoid-induced hyperglycaemia. A recent meta-analysis of 18 studies and 3642 COPD patients demonstrated that systemic glucocorticoid use (≥5 mg/day prednisolone equivalent for ≥3 days) was associated with a substantial increase in hyperglycaemia risk, with a pooled prevalence of 38.6% (95% CI: 29.9–47.9%) and a 2.4-fold higher likelihood of developing new-onset hyperglycaemia compared with non-users [69]. Further supporting these findings, a prospective study investigating COPD patients without pre-existing diabetes found that those who developed steroid-induced hyperglycaemia during acute exacerbations were at a markedly higher risk of future glucose metabolism disorders. Among hyperglycaemic patients, 40% developed prediabetes and 12% developed T2D during follow-up, whereas none of the normoglycemic patients did [70]. Even after adjusting for confounders such as BMI, hypertension, and corticosteroid dose, hyperglycaemic patients had a 37-fold higher risk of developing impaired glucose tolerance (*p* = 0.003) [70]. These findings suggest that steroid-induced hyperglycaemia in COPD may be an early marker of future diabetes risk, warranting close metabolic monitoring in this patient group.

Various pathophysiological mechanisms are responsible for the increased risk of glucocorticosteroid (GC)-induced insulin resistance and the development of T2D in these patients [71]. Tissues that are responsible for glucose homeostasis, such as the liver, skeletal muscle and adipose tissue, are affected by chronic hypercortisolism. In particular, chronic exposure to GCs stimulates hepatic gluconeogenesis by upregulating key enzymes such as phosphoenolpyruvate carboxykinase and glucose-6-phosphatase, while also enhancing lipolysis in adipose tissue and protein catabolism in skeletal muscle [72,73,74,75]. These processes increase the delivery of gluconeogenic substrates to the liver, promoting hepatic insulin resistance and fat accumulation [72,73,74,75]. Additionally, GCs impair pancreatic β-cell function, reducing insulin secretion. In skeletal muscle, GCs inhibit insulin signalling by downregulating IRS1, PI3K, and AKT, thereby reducing GLUT4 translocation and glucose uptake—a key contributor to GC-induced diabetes [76,77,78,79]. Although glucocorticoid-induced diabetes is a well-known complication of hypercortisolism, its prevalence is often underdiagnosed due to diagnostic variability and patient heterogeneity [80,81]. It is estimated that around 2% of new-onset diabetes cases worldwide are attributable to glucocorticoid exposure, underscoring the need for vigilant monitoring of glucose metabolism during corticosteroid therapy [82,83].

In conclusion, while corticosteroids remain indispensable for the management of acute COPD exacerbations, their use carries significant adverse metabolic consequences, particularly in patients with T2D. Clinicians should administer the lowest effective dose for the shortest duration possible, prioritize steroid-sparing approaches when feasible, and ensure rigorous glucose monitoring throughout treatment to minimize long-term metabolic harm in patients with COPD and coexisting T2D.

## 7. Clinical Implications

The coexistence of COPD and T2D is associated with more frequent exacerbations, prolonged hospitalizations, and higher mortality. Hyperglycaemia impairs immune function, increasing susceptibility to respiratory infections and worsening pulmonary outcomes [84]. Chronic systemic inflammation and oxidative stress further accelerate lung function decline, leading to more frequent exacerbations and a higher risk of respiratory failure [85]. Additionally, patients with COPD and poorly controlled diabetes exhibit reduced responsiveness to bronchodilators and delayed recovery after exacerbations [86].

Patients with both COPD and T2D have a markedly increased risk of CVD, including myocardial infarction, stroke, and heart failure [87]. The combination of systemic inflammation, oxidative stress, and chronic hypoxia induces cardiac remodelling and diastolic dysfunction, which increases the risk of heart failure with preserved ejection fraction (HFpEF). Additionally, hyperglycaemia, lipotoxicity, and insulin resistance accelerate myocardial fibrosis by stimulating cardiac fibroblasts to increase extracellular matrix production, trigger paracrine signalling in cardiomyocytes, immune cells, and vascular cells, promote the release of fibroblast-activating mediators and impair relaxation, contributing to HFpEF [88,89].

Additionally, the combination of pulmonary vasoconstriction due to chronic hypoxia and hyperglycaemia-induced vascular stiffness contributes to pulmonary hypertension and right ventricular dysfunction. Hyperglycaemia causes endothelial dysfunction from reduced NO bioavailability, leading to vasoconstriction and arterial stiffness, resulting in increased risk of stroke, hypertension, and ischemic heart disease [90,91].

Patients with comorbid COPD and T2D experience a substantial decline in quality of life due to overlapping symptoms such as fatigue, dyspnoea, and reduced exercise capacity [92]. These patients also exhibit increased healthcare utilization, including more frequent hospitalizations, emergency visits, and critical care admissions, often associated with more severe COPD exacerbations, prolonged recovery, and higher in-hospital mortality [93,94].

## 8. Therapeutic Strategies

### 8.1. Lifestyle

There are various lifestyle interventions that can be applied to this population. Tobacco use is a major modifiable risk factor for both COPD and T2D, contributing to disease progression, cardiovascular complications, and increased mortality, while longitudinal analyses demonstrate that smoking and COPD interact to influence body mass index trajectories, potentially contributing to metabolic dysfunction [95]. Effective smoking cessation requires a multifaceted approach that combines behavioural counselling, patient education, and pharmacological support, such as nicotine replacement therapy, bupropion, or varenicline [96]. Integrating smoking cessation interventions into routine clinical visits ensures consistent reinforcement, facilitates ongoing monitoring, and improves patient adherence [96,97]. Additionally, providing access to support groups, digital health tools, and follow-up counselling can enhance motivation, reduce relapse rates, and ultimately improve both respiratory and metabolic outcomes in patients with comorbid COPD and T2D [96,97].

Maintaining a balanced diet is essential for managing both COPD and T2D. Diets rich in whole grains, lean proteins, fruits, vegetables, and healthy fats, such as the traditional Mediterranean diet, support glycaemic control, reduce systemic inflammation, and help preserve muscle mass and pulmonary function [98,99,100]. Conversely, consumption of processed foods, refined carbohydrates, sugary beverages, and trans fats should be minimized due to their detrimental effects on metabolism and cardiovascular health. Tailored nutritional programmes, guided by registered dietitians and adapted to the individual’s metabolic, pulmonary, and lifestyle needs, can improve adherence to dietary recommendations, enhance overall health outcomes, and complement pharmacologic and lifestyle interventions [98,99,100]. Incorporating regular follow-ups, meal planning, and patient education further reinforces sustainable dietary habits and long-term disease management.

Regular physical exercise plays a critical role in managing both COPD and T2D by improving insulin sensitivity, enhancing pulmonary function, preserving muscle mass, and reducing systemic inflammation [101,102]. Recent consensus statements emphasize the importance of tailoring exercise prescriptions to COPD patients with relevant comorbidities, including metabolic disease [103]. Pulmonary rehabilitation programmes should combine aerobic exercises, such as walking or cycling, with strength and resistance training, tailored to each patient’s respiratory capacity and overall fitness level [101,102]. Collaboration with physiotherapists and rehabilitation specialists ensures that exercise routines are safe, effective, and progressive, minimizing the risk of exacerbations or injury. Incorporating structured exercise plans, ongoing supervision, and patient education on breathing techniques can optimize adherence, enhance functional capacity, and improve quality of life for patients with comorbid COPD and T2D [101,102].

### 8.2. Pharmacologic Interventions (Table 1)

Recent evidence suggests that glucose-lowering therapies such as GLP-1 receptor agonists and SGLT-2 inhibitors may exert pleiotropic anti-inflammatory and cardiopulmonary benefits in COPD beyond glycaemic control [104].

#### 8.2.1. Metformin

Metformin, a biguanide-class antihyperglycemic agent, remains the cornerstone of first-line pharmacological therapy for T2D and has recently gained attention for its potential benefits in patients with COPD [105]. It lowers blood glucose levels by suppressing hepatic glucose production, enhancing peripheral insulin sensitivity, and increasing the secretion of growth differentiation factor 15 (GDF15), which helps reduce appetite and caloric intake [105].

Beyond its glucose-lowering effects, emerging evidence suggests that metformin has pleiotropic actions on oxidative stress, inflammation, and cellular metabolism, pathways that are highly relevant in COPD pathophysiology. In a study investigating the effects of metformin in COPD and chronic cigarette smoke (CS) exposure in mice, metformin administration significantly attenuated CS-induced emphysema, oxidative stress, inflammation, and epithelial senescence [106]. Metformin activated AMPK and *GDF15* signalling, enhancing mitochondrial bioenergetics while reducing endoplasmic reticulum stress and unfolded protein responses [106]. In a different study, metformin was shown to protect against acute respiratory distress syndrome (ARDS), a severe inflammatory condition characterized by endothelial cell (EC) injury [107]. Using a lipopolysaccharide (LPS)-induced lung injury model, researchers found that metformin markedly reduced pulmonary inflammation and tissue damage by inhibiting endothelial pyroptosis. The treatment suppressed *NLRP3* inflammasome activation, decreasing cleaved caspase-1, GSDMD-N, IL-1β, and vascular adhesion molecule expression [107]. In cultured ECs, both metformin and the *NLRP3* inhibitor MCC950 attenuated LPS-induced pyroptosis [107]. In particular, metformin inhibited *NF-κB* activation and upregulated sirtuin-1 (*SIRT1*), while *SIRT1* inhibition reversed its protective effects. These findings suggest that metformin alleviates LPS-induced acute lung injury by suppressing *NF-κB*–*NLRP3*-mediated endothelial pyroptosis via *SIRT1* activation [107].

**Table 1:** Effects of antidiabetic drugs in patients with COPD.

Drug Class	Mechanism in T2D	Effects in COPD Patients	Risks/Cautions	References
**Metformin**	↓ hepatic glucose production ↑ insulin sensitivity ↑ *GDF15 *→ ↓ appetite and caloric intake	↓ oxidative stress, inflammation, epithelial senescence AMPK activation protection against ARDS via ↓ *NLRP3*-mediated pyroptosis and ↑ *SIRT1* may reduce COPD-related inflammation and lung tissue damage	Risk of lactic acidosis in severe hypoxaemia, advanced COPD or renal dysfunction Gastrointestinal intolerance Caution in frail or hypoxic patients	[106,107,108,109,110,111]
**GLP-1RAs**	↑ insulin secretion ↓ glucagon delayed gastric emptying promotes satiety and weight loss	↓ moderate and severe COPD exacerbations vs. sulfonylureas and DPP-4is possible anti-inflammatory or airway-protective effects ↓ mortality ↓ pneumonia ↓ ventilation need ↓ cardiovascular events ↑ FVC, DLCO	Gastrointestinal adverse effects (nausea, vomiting), risk of dehydration and weight loss Caution in frail, underweight, or malnourished patients	[112,113,114,115]
**DPP-4 Inhibitors**	↑ incretin activity → ↑ insulin, ↓ glucagon	↓ pulmonary inflammation ↓ oxidative stress ↓ immune cell recruitment modulation of *NF-κB*/*Nrf2* ↓ all-cause mortality ↓ COPD hospitalizations ↓ ventilation need ↓ bacterial pneumonia ↓ lung cancer risk modest ↓ cardiovascular events	Generally well tolerated Possible increased risk of infections	[116,117,118]
**Thiazolidinediones**	↑ insulin sensitivity ↑ adiponectin ↓ hepatic gluconeogenesis insulin-dependent glucose uptake in skeletal muscle and adipose tissue	Preclinical studies show anti-inflammatory and lung-protective effects ↓ endotoxin- and LPS-induced lung injury (↓ neutrophils, cytokines, oxidative stress) ↑ alveolar fluid clearance Possible reduced COPD exacerbations in observational studies	Fluid retention, weight gain, increased risk of heart failure and respiratory infections Caution in patients with cardiovascular disease or edema	[119,120,121,122,123,124]
**SGLT-2 Inhibitors**	↑ urinary glucose excretion mild weight loss ↓ blood pressure	↓ COPD exacerbations ↓ hospitalisations for COPD ↓ ventilation need ↓ all-cause mortality	Genital and urinary tract infections, volume depletion, hypotension Rare diabetic ketoacidosis and acute kidney injury Caution in elderly, dehydrated, or renally impaired patients	[11,12,125,126,127,128,129]

Abbreviations: COPD: Chronic Obstructive Pulmonary Disease, T2D: Type 2 Diabetes, GDF15: Growth differentiation factor 15, ARDS: Acute respiratory distress syndrome, AMPK: AMP-activated protein kinase, *NLRP3*: NLR family pyrin domain containing 3, *SIRT1*: Sirtuin 1, GLP-1RAs: Glucagon-like peptide-1 receptor agonists, DPP-4is: Dipeptidyl Peptidase-4 inhibitors, FVC: Forced Vital Capacity, DLCO: Diffusing capacity for carbon monoxide, *NF-κB*/*Nrf2*: Nuclear Factor kappa-light-chain-enhancer of activated B cells/Nuclear factor erythroid 2-related factor 2, LPS: Lipopolysaccharides, SGLT-2 Inhibitors: Sodium–Glucose Cotransporter-2 inhibitors, ↑ indicates increase/enhancement, ↓ indicates decrease/reduction.

From a clinical perspective, metformin is generally well tolerated in patients with stable COPD and T2D. Retrospective studies and systematic reviews indicate that while metformin may slightly elevate lactate levels, this increase is typically minor and not clinically significant in stable patients [108,109,110]. The primary risk arises in cases of severe hypoxia, renal impairment, or acute illness, where impaired lactate clearance can increase the likelihood of metformin-associated lactic acidosis (MALA) [111]. Consequently, careful patient selection, individualized risk assessment, and periodic monitoring of lactate and renal function are recommended to maximize glycaemic control benefits while minimizing potential adverse effects in COPD–T2D patients.

Despite promising experimental and observational findings, robust randomized clinical trial evidence demonstrating direct pulmonary benefits of metformin in patients with COPD is currently lacking, and its potential role beyond glycaemic control should therefore be considered exploratory.

#### 8.2.2. GLP 1 Receptor Agonists

GLP-1RAs (e.g., liraglutide, semaglutide) are a class of medications that mimics the mechanism of action of the incretin hormone GLP-1 [130]. They improve glucose homeostasis by activating GLP-1 receptors on pancreatic β-cells and neurons [130]. This activation enhances insulin secretion, suppresses glucagon release, and delays gastric emptying [130]. GLP-1RAs also promote satiety, reduce caloric intake, and facilitate weight loss, making them effective treatments for both T2D and obesity [130].

Recent studies suggest that GLP-1RAs may also have pulmonary benefits in patients with coexisting COPD and T2D. In a retrospective, electronic health record study of 1642 patients with COPD and T2D, initiation of GLP-1RAs was associated with a lower risk of both moderate and severe COPD exacerbations compared with sulfonylureas and dipeptidyl peptidase-4 inhibitors (DPP-4is), though no difference was found in comparison to sodium–glucose cotransporter-2 inhibitors (SGLT-2is) [112]. Even after adjusting for baseline glucose control, BMI, and metabolic variables, GLP-1RAs users exhibited fewer exacerbations, suggesting a potential anti-inflammatory or airway-protective effect independent of glycaemic control [112].

A nationwide study in Taiwan with 8060 matched patients with T2D and COPD used data from 2008 to 2019 to evaluate the impact of GLP-1RAs on cardiopulmonary outcomes [113]. Using Cox proportional hazards models, the study found that GLP-1RAs users had significantly lower risks of all-cause mortality [adjusted Hazard Ratio (aHR) 0.46, 95% CI 0.38–0.56], cardiovascular events (aHR 0.73, 95% CI 0.65–0.82), non-invasive ventilation (aHR 0.66, 95% CI 0.47–0.93), invasive mechanical ventilation (aHR 0.64, 95% CI 0.51–0.80), and bacterial pneumonia (aHR 0.76, 95% CI 0.65–0.88) compared with non-users [113]. Subgroup and duration analyses confirmed consistent protective associations across multiple outcomes [113]. Overall, the findings suggest that GLP-1RAs may have substantial cardiopulmonary and survival benefits in patients with coexisting T2D and COPD, supporting their potential role as a preferred therapeutic option in this high-risk population [113].

Furthermore, a randomized controlled, double-blind study that included 40 obese individuals with COPD showed that 40 weeks of treatment with liraglutide (3.0 mg) resulted in significant weight loss, increased FVC and diffusing capacity, and improved COPD assessment test scores versus placebo, although FEV_1_ and 6 min walking distance did not show significant change [114].

Finally, in a 2025 meta-analysis of six retrospective observational studies with 62,678 participants with T2D and COPD or asthma, GLP-1RA therapy was linked to a significantly lower risk of exacerbations compared to sulfonylureas [internal rate of return (IRR) = 0.52, 95% CI 0.42–0.64] and DPP-4is (IRR = 0.63, 95% CI 0.47–0.86), but not versus SGLT-2is [115]. In addition, in one study the exacerbation rate was lower among GLP-1RAs users compared with insulin users (IRR 0.39, 95% CI 0.26–0.58) but higher than in metformin users in a Japanese cohort (IRR 1.24, 95% CI 1.08–1.43).

Overall, these outcomes indicate that GLP-1RAs may provide metabolic, pulmonary, and survival benefits in patients with comorbid T2D and COPD, making them a promising therapeutic option in this population. As most available data are observational, causal inference is not possible and these findings should be interpreted cautiously pending confirmation in randomized controlled trials.

#### 8.2.3. DPP-4 Inhibitors

DPP-4is (e.g., sitagliptin, linagliptin) are oral antidiabetic agents approved for managing T2D in adults. These medications enhance incretin hormone activity, which helps regulate glucose homeostasis following food intake by increasing insulin secretion and suppressing glucagon release. Beyond their glucose-lowering effects, DPP-4is exhibit antihypertensive, anti-inflammatory, antiapoptotic, and immunomodulatory properties that benefit the heart, kidneys, and vasculature [131].

Preclinical studies have shown that DPP-4 inhibition reduces pulmonary inflammation, oxidative stress and vascular/remodelling responses in several models (LPS-induced lung injury, pulmonary hypertension, bleomycin/silica models). These effects are mediated via reduced proinflammatory cytokines, modulation of *NF-κB*/Nuclear factor erythroid 2-related factor 2 (*Nrf2*) pathways, and decreased immune cell infiltration [116,117].

In a large study utilizing data from Taiwan’s National Health Insurance Research Database (2008–2020), 55,924 pairs of propensity score-matched users and non-users of DPP-4 inhibitors with COPD and T2D were analyzed. Compared with non-users, DPP-4 inhibitor users demonstrated significantly reduced risks for all-cause mortality (aHR 0.47, 95% CI 0.45–0.49), COPD hospitalization (aHR 0.73, 95% CI 0.62–0.85), invasive mechanical ventilation (aHR 0.76, 95% CI 0.71–0.82), bacterial pneumonia (aHR 0.73, 95% CI 0.70–0.76), and lung cancer (aHR 0.74, 95% CI 0.71–0.78) [118]. A modest reduction in major adverse cardiovascular events (MACEs) was also observed (aHR 0.92, 95% CI 0.88–0.95) [118]. Kaplan–Meier analyses further confirmed a significantly lower cumulative incidence of COPD hospitalization, respiratory failure, lung cancer, bacterial pneumonia, and mortality among DPP-4is users (log-rank *p* ≤ 0.004) [118].

These findings suggest that DPP-4is therapy may provide multisystem protective benefits, including improved respiratory and survival outcomes, in patients with comorbid COPD and T2D. However, as most of these data originate from observational studies, these associations should not be interpreted as proof of causality, and residual confounding cannot be excluded.

#### 8.2.4. Thiazolidinediones

Thiazolidinediones (TZDs) (e.g., rosiglitazone) are insulin-sensitizing agents that modulate intracellular metabolic pathways to enhance insulin action and improve sensitivity in key peripheral tissues [132,133]. They elevate circulating adiponectin levels, suppress hepatic gluconeogenesis, and promote insulin-dependent glucose uptake in skeletal muscle and adipose tissue [132,133].

Preclinical studies showed that TZDs such as rosiglitazone and pioglitazone exert anti-inflammatory and lung-protective effects. Rosiglitazone reduces endotoxin- and LPS-induced lung injury by limiting neutrophil infiltration, oxidative stress, and pro-inflammatory cytokine release, while enhancing alveolar fluid clearance via ENaC/SGK1 pathways [119,120]. Similarly, pioglitazone mitigates bleomycin-induced lung inflammation and fibrosis by suppressing TNF-α and macrophage infiltration. Overall, TZDs demonstrate potential to reduce pulmonary inflammation and oxidative injury, supporting their possible therapeutic role in COPD [119].

Observational human studies suggest that TZDs may be associated with a modest reduction in COPD exacerbations compared with other antidiabetic drugs [121,122]. However, these findings are limited by potential confounding factors, such as disease severity and comorbidities. Moreover, some cohort studies have reported increased risks of pneumonia, and heart failure with TZD use, reflecting potential safety concerns in this population [123,124]. All things considered, while TZDs show promise for reducing pulmonary inflammation and exacerbations in COPD, current evidence remains largely preclinical or observational, underscoring the need for randomized controlled trials to clarify their therapeutic role and safety profile in patients with COPD and T2D.

#### 8.2.5. SGLT-2 Inhibitors

SGLT-2is function by blocking glucose reabsorption in the proximal renal tubules, leading to increased urinary glucose excretion, mild weight reduction, and lower blood pressure [125]. Clinical evidence has shown their efficacy in improving outcomes for heart failure, both with reduced and preserved ejection fraction, and in chronic kidney disease, leading to their inclusion in major international treatment guidelines [125].

**Figure 2:**
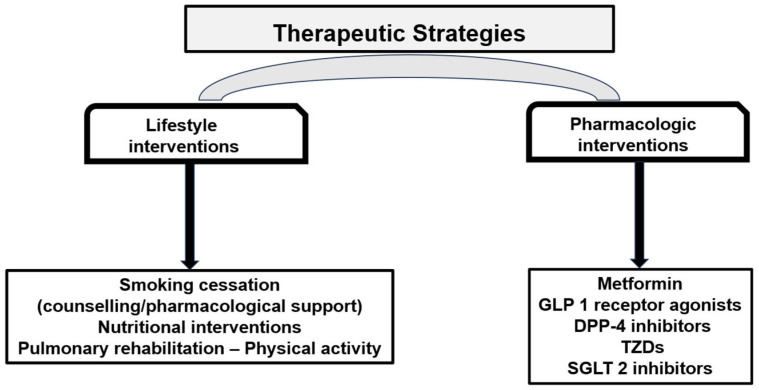
Therapeutic strategies: Lifestyle and pharmacologic interventions. Abbreviations: GLP 1 receptor agonists: Glucagon-like peptide-1 receptor agonists, DPP-4 inhibitors: Dipeptidyl Peptidase-4 inhibitors, TZDs: Thiazolidinediones, SGLT-2 inhibitors: Sodium–glucose cotransporter-2 inhibitors.

Meta-analyses and large observational cohort studies suggest that use of SGLT-2is in patients with COPD and T2D is associated with a reduced risk of COPD exacerbations, hospitalizations for COPD, ventilatory support (non-invasive or invasive), and all-cause mortality [11,126,127,128]. For example, one large cohort (299,168 patients) found aHR ≈ 0.79 (95% CI 0.67–0.93) for a composite endpoint of COPD hospitalisation/Non-Invasive Positive Pressure Ventilation/invasive ventilation/mortality in SGLT-2is users vs. non-users [11]. Comparative research also indicates that SGLT-2is may have greater protective effect against exacerbations compared to DPP-4is and perhaps similar or modestly better effect compared to GLP-1RAs [129]. Figure 2 summarizes therapeutic strategies, including lifestyle and pharmacologic interventions.

Although these findings are encouraging, they are largely based on observational cohorts and require confirmation in adequately powered randomized controlled trials specifically addressing COPD-related outcomes.

#### 8.2.6. Safety Considerations and Patient Selection

The selection of antidiabetic therapy in patients with comorbid COPD and T2D should carefully balance potential metabolic and pulmonary benefits against safety considerations. Metformin should be used with caution in patients with severe hypoxaemia, severe COPD, or severe renal dysfunction because of the rare but serious risk of lactic acidosis [108,110,111]. GLP-1 receptor agonists may be limited by gastrointestinal intolerance and unintended weight loss in frail or sarcopenic patients [114]. Thiazolidinediones may be associated with an increased risk of fluid retention, heart failure, and respiratory infections, particularly in patients with pre-existing cardiovascular disease, and thus should be avoided in patients with severe heart failure [123,124]. SGLT-2 inhibitors require careful monitoring in patients at risk of volume depletion and hypotension, diabetic ketoacidosis, or recurrent genitourinary infections [12,125]. Overall, treatment selection should take into account comorbidities, cardiovascular and renal status, nutritional condition, and infection risk, underscoring the need for individualized therapeutic strategies in this high-risk population.

## 9. Multidisciplinary Management and Future Directions for Patients with COPD and Type 2 Diabetes

Effective management of patients with coexisting COPD and T2D requires a comprehensive, multidisciplinary approach that integrates expertise from pulmonologists, endocrinologists, primary care providers, nutritionists, and rehabilitation specialists. Such coordinated care ensures that both metabolic and respiratory aspects of the patient’s health are addressed, optimizing outcomes and minimizing complications. Central to this approach are shared decision-making and patient education, empowering patients to actively participate in treatment planning, adhere to therapy, and adopt lifestyle modifications.

Despite emerging evidence regarding the potential benefits of antidiabetic therapies, such as metformin, GLP-1RAs, and SGLT-2is, in patients with COPD, substantial knowledge gaps remain. There is a pressing need for large, well-powered randomized controlled trials (RCTs) specifically designed to assess COPD-related endpoints, including exacerbation rates, lung function, and quality of life. Moreover, understanding phenotypic variability, for example, differences in airway inflammation, obesity, or metabolic profiles, could allow for personalized treatment strategies that maximize therapeutic benefit while minimizing adverse effects. Finally, the field would benefit from the standardization of definitions and outcome measures across studies of metabolic syndrome and COPD, facilitating comparisons between trials and improving the translation of research findings into clinical practice. Collectively, these strategies highlight the importance of integrated, evidence-based, and patient-centred care in this high-risk population.

## 10. Conclusions

Comorbidity of COPD and type 2 diabetes is prevalent and is associated with adverse clinical outcomes. A growing body of evidence highlights shared pathophysiological mechanisms, including systemic inflammation, oxidative stress, hypoxia, and metabolic dysregulation, which contribute to disease progression and multimorbidity. An integrated management approach, including lifestyle modifications and tailored pharmacologic therapies, may improve both pulmonary and metabolic outcomes in this high-risk population. However, most available evidence is observational, and additional well-designed randomized clinical trials are needed to define optimal treatment strategies and to establish the clinical impact of emerging antidiabetic therapies in patients with coexisting COPD and T2D.

## Data Availability

No new data were created or analyzed in this study.

## Abbreviations

The following abbreviations are used in this manuscript:

COPD	Chronic Obstructive Pulmonary Disease
MetS	Metabolic Syndrome
CVD	Cardiovascular Disease
T2D	Type 2 Diabetes
GLP-1RAs	Glucagon-Like Peptide-1 Receptor agonists
SGLT-2is	Sodium-Glucose Cotransporter-2 inhibitors
MeSH	Medical Subject Heading
OR	Odds Ratio
CI	Confidence Interval
RR	Relative Risk
FVC	Forced Vital Capacity
FEV_1_	Forced Expiratory Volume In 1 Second
AECOPD	Acute Exacerbations of COPD
TNF-α	Tumour Necrosis Factor-alpha
IL-6	Interleukin-6
CRP	C-reactive protein
IRS-1	Insulin Receptor Substrate-1
FFAs	Free Fatty Acids
NO	Nitric Oxide
*NF-κB*	Nuclear Factor-kappa B
*NLRP3*	NLR family pyrin domain containing 3
ROS	Reactive Oxygen Species
AGEs	Advanced Glycation End-Products
RAGE	Receptor For Advanced Glycation End Products
*HIF-1α*	Hypoxia-Inducible Factor-1α
SB	Sedentary Behaviour
METs	Metabolic Equivalents
PA	Physical Activity
*CPT1B*	Carnitine Palmitoyltransferase 1B
NADPH	Nicotinamide Adenine Dinucleotide Phosphate Oxidase
SHBG	Sex hormone-binding globulin
Glut-4	Glucose transporter type 4
SO	Sarcopenic obesity
AMPK	AMP-activated protein kinase
MR	Mendelian randomization
SNPs	Single Nucleotide Polymorphisms
GC	Glucocorticosteroid
HFpEF	Heart Failure with preserved Ejection Fraction
GDF15	Growth Differentiation Factor 15
CS	Cigarette Smoke
ARDS	Acute Respiratory Distress Syndrome
EC	Endothelial Cell
LPS	Lipopolysaccharide
DLCO	Diffusing capacity for carbon monoxide
MALA	Metformin-Associated Lactic Acidosis
DPP-4is	dipeptidyl peptidase-4 inhibitors
aHR	adjusted Hazard Ratio
*Nrf2*	Nuclear factor erythroid 2-related factor 2
IRR	Internal Rate of Return
MACEs	Major Adverse Cardiovascular Events
TZDs	Thiazolidinediones
RCTs	Powered Randomized Controlled Trials
